# Breastfeeding, HIV exposure, childhood obesity, and prehypertension: A South African cohort study

**DOI:** 10.1371/journal.pmed.1002889

**Published:** 2019-08-27

**Authors:** Brian Houle, Tamsen J. Rochat, Marie-Louise Newell, Alan Stein, Ruth M. Bland

**Affiliations:** 1 School of Demography, The Australian National University, Canberra, Australia; 2 MRC/Wits Rural Public Health and Health Transitions Research Unit (Agincourt), School of Public Health, Faculty of Health Sciences, University of the Witwatersrand, Johannesburg, South Africa; 3 CU Population Center, Institute of Behavioral Science, University of Colorado at Boulder, Boulder, Colorado; 4 Africa Health Research Institute, KwaZulu-Natal, South Africa; 5 MRC/Developmental Pathways to Health Research Unit, Department of Paediatrics, School of Clinical Medicine, Faculty of Health Sciences, University of Witwatersrand, South Africa; 6 School of Human Development and Health, Faculty of Medicine, University of Southampton, Southampton, United Kingdom; 7 School of Public Health, Faculty of Health Sciences, University of Witwatersrand, Johannesburg, South Africa; 8 Section of Child and Adolescent Psychiatry, Department of Psychiatry, University of Oxford, United Kingdom; 9 Institute of Health and Wellbeing and Royal Hospital for Children, University of Glasgow, Glasgow, United Kingdom; Cornell University, UNITED STATES

## Abstract

**Background:**

Evidence on the association between breastfeeding and later childhood obesity and blood pressure (BP) is inconsistent, especially in HIV-prevalent areas where, until recently, HIV-infected women were discouraged from breastfeeding, but obesity is increasingly prevalent.

**Methods and findings:**

The Siyakhula cohort (2012–2014), a population-based prospective cohort study, collected data over 3 visits on HIV-negative children ages 7 to 11 years in rural South Africa. We used weight (body mass index [BMI]), fat, and BP as outcome variables and incorporated early life (including mother’s age at delivery and HIV status) and current life factors (including maternal education and current BMI). Our primary exposure was breastfeeding duration. We dichotomized 3 outcome measures using pre-established thresholds for clinical interpretability: (1) overfat: ≥85th percentile of body fat; (2) overweight: >1 SD BMI z score; and (3) prehypertension: ≥90th percentile for systolic BP (SBP) or diastolic BP (DBP). We modelled each outcome using multivariable logistic regression, including stopping breastfeeding, then early life, and finally current life factors. Of 1,536 children (mean age = 9.3 years; 872 girls; 664 boys), 7% were overfat, 13.2% overweight, and 9.1% prehypertensive. Over half (60%) of the mothers reported continued breastfeeding for 12+ months. In multivariable analyses, continued breastfeeding between 6 and 11 months was associated with approximately halved odds of both being overfat (adjusted odds ratio [aOR] = 0.43, 95% confidence interval [CI] 0.21–0.91, *P* = 0.027) and overweight (aOR = 0.46, CI 0.26–0.82, *P* = 0.0083), but the association with prehypertension did not reach statistical significance (aOR = 0.72, CI 0.38–1.37, *P* = 0.32). Children with a mother who was currently obese were 5 times more likely (aOR = 5.02, CI 2.47–10.20, *P* < 0.001) to be overfat and over 4 times more likely to be overweight (aOR = 4.33, CI 2.65–7.09, *P* < 0.001) than children with normal weight mothers. Differences between HIV-exposed and unexposed children on any of the outcomes were minimal and not significant. The main study limitation was that duration of breastfeeding was based on maternal recall.

**Conclusions:**

To our knowledge, this is the first study examining and quantifying the association between breastfeeding and childhood obesity in an African setting with high HIV prevalence. We observed that breastfeeding was independently associated with reduced childhood obesity for both HIV-exposed and unexposed children, suggesting that promoting optimal nutrition throughout the life course, starting with continued breastfeeding, may be critical to tackling the growing obesity epidemic. In the era of widespread effective antiretroviral treatment for HIV-infected women for life, these data further support the recommendation of breastfeeding for all women.

## Abstract 2

Brian Houle and colleagues reveal a protective effect against childhood obesity when mothers breastfeed.

## Author summary

**Why was this study done?:**

- Mounting evidence suggests a protective association between being breastfed and later obesity, mostly from resource-rich settings where HIV is not highly prevalent.
- However, data are inconsistent, with methodological problems including confounding with socioeconomic status (SES), which may influence both breastfeeding and later childhood obesity, small studies with the potential to overestimate the effects of breastfeeding, and a lack of studies allowing for maternal HIV status (HIV-infected women were, until recently, discouraged from breastfeeding because of concerns about postnatal transmission to the child).
- To our knowledge, this is the first study examining breastfeeding and childhood obesity in an African setting with high HIV rates where many women chose and were supported to breastfeed.

**What did the researchers do and find?:**

- We examined the association between breastfeeding, HIV exposure, and later childhood obesity, overfatness, and blood pressure (BP) in a large cohort of rural South African children.
- We found that breastfeeding beyond 6 months was associated with half the odds of both being overfat and overweight in children aged 7 to 11 years.
- Differences between children of HIV-positive and HIV-negative mothers in terms of their body fat, body mass index (BMI), and BP were small and not statistically significant.

**What do these findings mean?:**

- We provide evidence confirming that public health promotion of optimal nutrition through breastfeeding in early life has benefits that extend into midchildhood, for both children exposed to maternal HIV infection and those not exposed.
- Our findings suggest that, with the advent of widespread effective antiretroviral treatment for all HIV-infected pregnant women for life, which negates mother-to-child transmission risk, breastfeeding guidance could be considered for all women irrespective of maternal HIV status.
- This is especially pertinent as obesity levels and the number of HIV-exposed children continue to increase in many African settings.
- Future studies using prospective information on breastfeeding may be able to further clarify the protective effects of long-term breastfeeding on later child outcomes and explore the importance of when and what types of other foods or liquids are introduced into children’s diets.

## Introduction

Prevention and treatment of noncommunicable diseases (NCD), such as obesity and hypertension, to reduce premature mortality is a critical target of the Sustainable Development Goals [1]. Approximately 70% of premature deaths in 2013 were attributed to NCDs [2], and an estimated 82% of premature deaths occurred in low- and middle-income countries (LMIC) [3]. Adopting a life course approach to NCD prevention, the importance of early life exposures, including optimal nutrition for the child in the first 1,000 days of life, the onset of risk factors in childhood and adolescence, and their links to NCDs in later life have been highlighted [4–6].

Evidence linking breastfeeding and later risk of obesity and hypertension is inconsistent [7–18], due to heterogeneity in study findings and differences in the way breastfeeding is defined and measured. A recent systematic review [19], including 105 studies (34 from LMIC), updated evidence on the long-term benefits of breastfeeding across a range of age groups and found an inverse association between breastfeeding (using several different categorizations) and being overweight, with no significant effect on blood pressure (BP). However, methodological problems including confounding by socioeconomic status (SES) were noted, with most studies from high-income countries where breastfeeding was more prevalent among wealthier mothers, and publication bias in which the effects of breastfeeding were overestimated in small studies. In many resource-limited settings, HIV has complicated infant feeding decisions [20], with breastfeeding avoided or curtailed. Around the turn of the century in South Africa, the prevalence of HIV among pregnant women was up to 40% [21], with prevailing clinical guidelines discouraging these women from breastfeeding, or if they did breastfeed, to stop no later than at 6 months. In many South African provinces, formula milk was provided free of charge to pregnant women, encouraging them to avoid breastfeeding and also undermining breastfeeding rates in women who were HIV-uninfected or of unknown HIV status [22]. In the study area, provincial health policy was to counsel HIV-infected women individually on infant feeding choices with support for 6 months exclusive breastfeeding for those choosing not to formula feed, while promoting breastfeeding in the general population. Little is known about the long-term benefits of breastfeeding in HIV-endemic regions, many of which currently also face an adult obesity crisis [23]. In this study, we have the opportunity to explore the long-term effects of breastfeeding in a population where breastfeeding was promoted and supported.

In sub-Saharan Africa, the increasing success of prevention of mother-to-child transmission (PMTCT) programs has considerably reduced the number of HIV-infected infants but increased the number of HIV-exposed, uninfected children [24]. Evidence indicates that compared with children of HIV-uninfected women, children born to HIV-infected women have lower birth weights [25–28] and are more likely to be small for gestational age [29]. Larger cohorts have shown normal subsequent growth of HIV-exposed compared with HIV-unexposed children [30–32]. A few smaller studies have suggested that HIV-exposed children have lower weights during childhood than HIV-unexposed children [33,34], although a study in Zambia reported that HIV-exposed children showed less decline in weight-for-age z scores between 4.5 and 15 months if they were breastfed [35]. We have demonstrated, in a large cohort breastfeeding study in South Africa, the Vertical Transmission Study (VTS), that for children aged up to 24 months, HIV-exposed children grew as well as the reference population [32]. Given the increasing number of HIV-exposed children and increasing obesity levels in HIV-prevalent settings, assessment of the role of breastfeeding in their short- and long-term outcomes is critical [24].

We explore 3 child risk factors for later NCDs: overweight, overfat, and prehypertension [36–38] measured at ages 7 to 11 years in a cohort of primary school-aged children in the context of HIV in rural South Africa. South Africa represents an important setting to understand the role of early life nutrition and later NCD risk, given a continued HIV epidemic alongside a persistent burden of childhood undernutrition and increasing obesity levels and their links to later NCDs [39–42]. Results from the 2016 South African Demographic and Health Survey showed that 6% of children under 5 years old were underweight and 13% were overweight [43]; estimated continued breastfeeding to 12 months was over 50% [43]. A cross-sectional study of South African children aged 8 to 11 years found a prevalence of approximately 4% for children under the third percentile for body mass index (BMI) and a prevalence of 27% for children above the 95th percentile [44]. Programmatic data from a large, postnatal clinic in Johannesburg, South Africa, described self-reported breastfeeding practices of 1,913 women at 6 months post partum: 58% of HIV-negative women reported that they had exclusively breastfed their infant compared with 37% of HIV-positive women [45].

We describe the prevalence of these chronic disease risk factors by breastfeeding duration and the association of sustained breastfeeding on these outcomes in a multivariable framework. We also assess differences in breastfeeding rates or effects by HIV exposure. We hypothesized that breastfeeding would result in lower odds of obesity, overfat, and prehypertension in childhood.

## Methods

Ethics permission was granted by the Biomedical Research Ethics Committee, University of KwaZulu-Natal, South Africa (BF184/12). This study is reported as per the Strengthening the Reporting of Observational Studies in Epidemiology (STROBE) guideline (S1 Checklist). The Siyakhula cohort was established in 2012 at the Africa Health Research Institute (AHRI; formerly the Africa Centre for Population Health), in rural KwaZulu-Natal, South Africa, including 7 to 11 year-old HIV-negative children of HIV-positive and HIV-negative mothers [46]. AHRI operates a demographic surveillance system (DSS) in 11,000 homes twice yearly [47], with data collected including births, deaths, SES, and adult HIV status. Children in the Siyakhula cohort were from 2 sources as has been previously described in detail by Rochat and colleagues [46]. Approximately half of the cohort children had participated (from birth to age 2 years) in a breastfeeding study, the VTS (2001–2006) [48]. The primary aim of the VTS was to determine the effect of infant feeding practices on vertical transmission of HIV infection; mothers were supported to exclusively breastfeed. The results demonstrated that exclusive breastfeeding was associated with a reduced risk of mother-to-child transmission of HIV compared with mixed breastfeeding [48,49]. The second group of children were enrolled from the DSS; these children were born during the same VTS time period but did not participate in the VTS and thus had not been exposed to infant feeding support. It is important to note that all eligible children within the DSS were approached for inclusion in the Siyakhula cohort—some of whom had participated in the VTS and others who had not. The Siyakhula cohort predated antiretroviral treatment roll-out in the country. It thus allows for examination of outcomes associated with HIV exposure without antiretroviral treatment (ART) exposure (which is associated with potential neurotoxicity and neurodevelopmental consequences in early childhood [50]) in utero and during breastfeeding. Early life data are available for all Siyakhula children from either the DSS, VTS, or both. Data from the DSS includes factors routinely collected during biannual collection rounds, and information from the VTS includes information collected during the 2 years of data collection [47,48]. Available early life data includes birth order, birth weight, mother’s age at birth, and mother’s HIV status. Information on child anthropometry were collected with the aim of examining the effect of early life factors on later outcomes [46]; we did not have a specific analysis plan prior to data collection. All research at AHRI was conducted with permission from the local health authorities. Any participants requiring medical care were referred to the local clinics or the hospital in the subdistrict that had a paediatric ward.

### Data collection

Data were collected over 3 visits between September 2012 and September 2014. After an initial telephone or home contact, women who expressed interest in the study were visited by a field worker who provided study details and obtained written informed consent (visit 1). At the second visit, fieldworkers collected data on current SES and health (including mother and child current HIV status) and documented mothers’ anthropometric measurements. Children’s anthropometry and BP were assessed at the third visit.

#### Child anthropometry and BP

All outcome measurements (height, weight, BP, and % body fat estimates) were carried out by field workers and standard operating procedures were in adherence with World Health Organization (WHO) standards [51,52]. All measurements were carried out twice. Any measurement falling outside the maximum allowed difference was repeated, and the 2 measurements within the allowed differences were recorded. Equipment was calibrated every month, and quality assurance checks were conducted by the study manager throughout the study.

Height was measured to the nearest 0.1 cm using a SECA scale stadiometer. If the difference between the 2 measurements was greater than 5 mm, a third measurement was taken, and the 2 heights within 5 mm were recorded.

Weight and body fat were measured using the TANITA SC240MA bio-impedance digital scales, with indoor clothing on (shoes and socks removed). Weight was measured to the nearest 0.1 kg and body fat estimated to the nearest 0.1%. On the TANITA device, “non-athlete” was chosen as the standard mode, and 0.5 kg was entered as the standard deduction for clothes’ weight. The TANITA device allows for sex, age, and height. For weights, if the 2 recorded weights had a difference greater than 100 g, a third measurement was taken, and the 2 weights within 100 g were recorded. BMI was calculated (kg/m^2^) from an individual’s mean weight and height.

BP was measured using an automated BP monitor (A&D Medical, Model UA-767 Plus 30) and appropriately sized cuff, with the child seated on a chair for at least 5 minutes and prior to the cognitive assessments (which were carried out in this cohort and reported on elsewhere [53]). Systolic blood pressure (SBP) and diastolic blood pressure (DBP) levels were taken twice and if the difference in either measure was greater than 5 mm, a third measurement was taken, and the 2 measurements within 5 mm used.

#### Maternal anthropometry

Weight and height measurements followed the same methods as for the children using the same equipment. For BP, an adult cuff was used.

#### Breastfeeding

Data on all children were collected by maternal recall at the time of the Siyakhula visit. Each mother was asked when she stopped all breastfeeding (for assessment of total breastfeeding duration). Although the VTS collected data on breastfeeding at weekly intervals, because this is not available for the children enrolled from the DSS, all data on breastfeeding used for these analyses were collected by maternal recall.

#### HIV exposure

Maternal HIV status was defined as “positive” for those positive during pregnancy (children HIV-exposed); “negative” for those who have remained HIV-negative since delivery (children HIV-unexposed); and “positive since” for those who were HIV-negative during pregnancy and subsequently acquired HIV (children HIV-affected).

### Child outcome measures

We examined 3 child risk factors for later NCDs: overweight (including obese), overfat (including obese-fat), and prehypertension (including hypertension). A mean was calculated from the 2 measurements for each assessment of height, weight, and BP. We chose to measure both overweight (based on BMI) and overfat (based on body fat measures), with references previously used in this study area [54]. In the absence of other applicable body fat references, we used the McCarthy (2006) references for body fat [55], which relate to Caucasian children only. However, we have previously shown that simple anthropometric methods used to define weight status (e.g., BMI) in children in this population produced lower estimates of unhealthy weight status than those derived from body fatness measures [54]. Therefore, we included both measures in the analyses.

#### Overweight

Anthroplus software was used for application of WHO Reference 2007 for children aged 5 to 19 years [56,57]. The International Obesity Taskforce (IOTF) approach was used to define overweight and obesity, defining BMI-for-age as z score of >+1SD, conceptually equivalent to a BMI at age 18 of ≥25 kg/m^2^ for overweight and obese [58].

#### Overfat

Body fat estimates from bio-impedance were categorized into underfat, healthy, and overfat (including obese-fat), by age and sex, using McCarthy and colleagues [55] 2006 body fat reference curves for children. Overfatness was defined for children and adolescents based on bio-impedance (TANITA SC240MA), using cut-offs for excess fatness that were age and sex specific and defined as the 85th percentile of body fat percent from the McCarthy reference [55].

#### Prehypertension and hypertension

Mean SBP and DBP were categorized into normal and prehypertensive and hypertensive using height z scores to compute age- and sex-specific SBP and DBP z scores, which were then transformed into percentiles based on the National High Blood Pressure Education Program Working Group on Children and Adolescents guidelines [59].

We dichotomized each outcome according to pre-established thresholds to increase interpretability in terms of clinical risks: (1) overfat (including obese-fat) at ≥85th percentile of body fat; (2) overweight (including obesity) at >1 SD BMI z score; and (3) prehypertension (including hypertension) at ≥90th percentile for either SBP or DBP.

### Statistical analyses

We first calculated the prevalence of each child outcome by age when the child stopped receiving any breastmilk—categorized as less than 1 month, 1 to 5 months, 6 to 11 months, and 12 or more months. We chose these categories based on breastfeeding guidelines at the time [60,61] and examination of the recall data, which indicated substantial data heaping at 6 and 12 months. As most women report to start breastfeeding, less than 1 month captures both those who never breastfed and those who breastfed only briefly. The category of 1 to 5 months captures those who did not reach the 6-month recommended target for exclusive breastfeeding. At the time, HIV-infected women were counselled to stop breastfeeding completely at 6 months, but continued breastfeeding was advised for non-HIV-infected women, captured by the category of 6 to 11 months. Finally, as many women stop breastfeeding in the first year, the category of 12 or more months captures those who sustained breastfeeding into the second year of life.

We next modelled each outcome using complete case logistic regression, adjusting for intramother correlation for children with the same mother. Our selection of early and current life covariates were based on existing literature and conceptual reasoning and included factors related to birth, demographics, maternal anthropometry, and socioeconomic factors. First, we modelled each outcome including breastfeeding duration only. Next, we included early life factors: birth order (categorized as 1–2, 3–4, and 5+), birthweight (<2.5 kg and ≥2.5 kg), and mother’s age at birth (<20, 20–29, and 30+ years) and HIV status (negative, positive pregnancy, positive since pregnancy). Lastly, we included current life factors: any overnight child hospitalizations since birth (as a measure of the child being unwell or having a chronic condition that may affect the outcomes), and mother’s current education (none/primary and some secondary or higher) and BMI (categorized as underweight [<18.5 kg/m^2^], normal [18.5–24 kg/m^2^], overweight [25–29 kg/m^2^], and obese [≥30 kg/m^2^]), and whether the household owned a fridge (as a proxy for SES; based on a principal component analysis to identify the variables explaining the overall variance). For overfat, we also included if the child was stunted (≤2 SD height z score) in the final model. To assess for the potential of differences in breastfeeding rates or effects by HIV exposure, we tested the final model of each outcome including an interaction between breastfeeding and HIV exposure (comparing exposed to unexposed (collapsing HIV-unexposed and affected children together)). As each outcome accounts for child sex and age, these variables were not included in the models. We summarize our models using predictive probabilities based on the model estimates, allowing each covariate to be at its observed value. We completed all analyses using Stata 15 [62].

As an assessment of the accuracy of breastfeeding recall, for the subsample of VTS children for whom recall and prospective feeding data were available, we compared the proportions of children whose breastfeeding duration were accurately recalled according to our categorizations. As a test of the robustness of our results, we also re-estimated the final model for each outcome on the VTS subsample only using breastfeeding duration based on prospective feeding data.

We explored several other approaches to modelling the association of breastfeeding with the 3 outcomes. We fit a series of models treating the outcomes as continuous (using quantile regression), selecting the quantile for each outcome based on the percentage of observations at or below each of the clinical thresholds as per above [63,64]. We also included models on underfat and underweight based on the percentage of observations at or below clinical thresholds of the age and sex specific second percentile for body fat percent [55] and ≤−2 SD for BMI z score [54]. In these continuous models, we included child sex and age in the overfat and underfat models because the continuous data are not sex or age adjusted for these outcomes.

## Results

**Fig 1:**
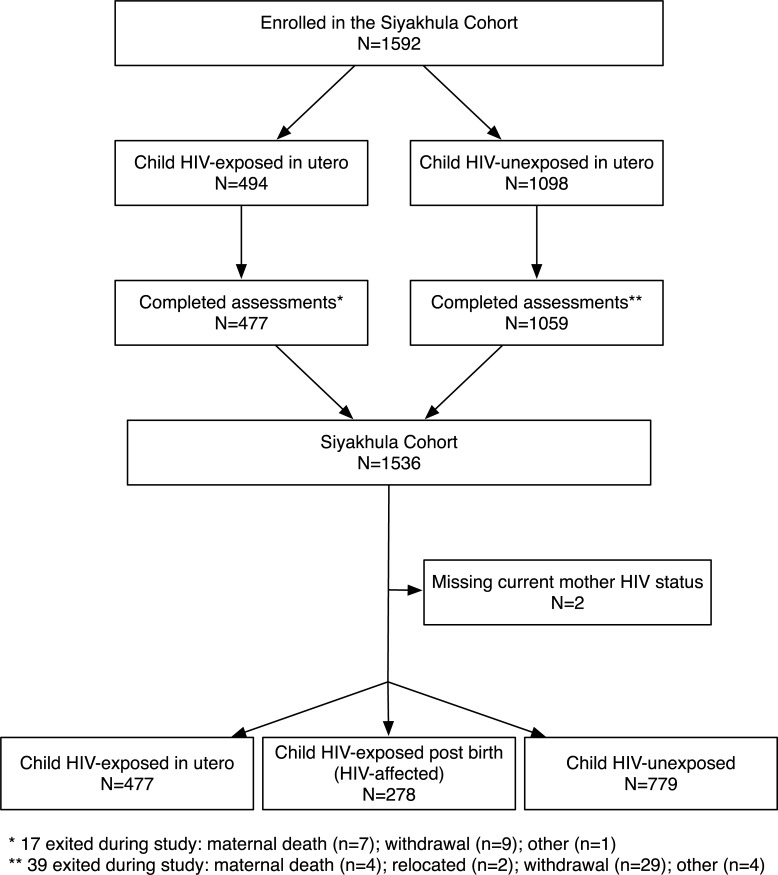
Participant flowchart for the Siyakhula cohort by children’s HIV exposure status.

Fig 1 shows the participant flowchart of the Siyakhula cohort: 1,592 of a possible 2,515 children were enrolled; 1,536 children completed assessments (477 HIV-exposed, 1,057 HIV-unexposed (278 HIV-affected)); the mothers of 2 children had missing HIV status. Detailed comparisons of eligible, enrolled, and completed subgroups are presented elsewhere [46].

At the time of the visit, child mean age was 9.3 years (range 7–11) and maternal age 36.0 years. Table 1 shows anthropometry and the NCD risk factors by breastfeeding duration. Twelve percent of the sample breastfed for less than 1 month, 6% for 1 to 5 months, 22% for 6 to 11 months, and 60% for 12+ months (1 child had missing breastfeeding duration). Compared with HIV-exposed children, HIV-unexposed children were more likely to be breastfed for 12 or more months. Overall, the prevalence of stunting was very low (3%). Approximately half of the sample had normal body fat measurements, over a third were underfat, and 7% were overfat or obese-fat (with 5% being obese-fat) and 13% overweight based on BMI. Prehypertension (including hypertension) prevalence was 9%, with nearly half of those being hypertensive (4.2%). Of the children who were overfat, overweight, or prehypertensive, 17% had 2 of these risk factors and 3% had all 3 risk factors. Obese-fat and overweight were more common among children breastfed for less than 1 month than children breastfed for 1 or more months. There was no association between fridge ownership (as an SES proxy found to be relevant in this setting) and breastfeeding duration.

**Table 1:** Prevalence of child anthropometry, NCD risk factor outcomes, and early life and current factors, by breastfeeding duration, child ages 7 to 11 years (*n* = 1,535).

	<1 mo, %	1–5 mo, %	6–11 mo, %	12+ mo, %	*p*-value	Total, %
**Height for age**	*n* = 177	*n* = 99	*n* = 343	*n* = 913	0.83	*n* = 1,532
>−2 SD	97.7	98.0	97.4	96.8		97.1
≤−2 SD	2.3	2.0	2.6	3.2		2.9
**Body fat**	*n* = 177	*n* = 99	*n* = 343	*n* = 912	0.026	*n* = 1,531
Underfat^a^	27.1	30.3	33.2	38.3		35.3
Normal	61.6	64.6	61.5	54.8		57.7
Overfat	2.8	0.0	1.7	2.1		2.0
Obese-fat	8.5	5.1	3.5	4.8		5.0
**BMI**	*n* = 176	*n* = 98	*n* = 341	*n* = 910	0.013	*n* = 1,525
≤−2 SD	1.1	1.0	2.9	5.1		3.9
−2 to 1 SD	79.5	84.7	85.3	82.5		83.0
>1 SD	19.3	14.3	11.7	12.4		13.2
**Prehypertension**	*n* = 177	*n* = 99	*n* = 343	*n* = 911	0.79	*n* = 1,530
Normal	88.1	92.9	91.3	91.1		90.9
Prehypertensive	7.3	4.0	4.4	4.7		4.9
Hypertensive	4.5	3.0	4.4	4.2		4.2
**Birth order**	*n* = 177	*n* = 99	*n* = 344	*n* = 915	0.018	*n* = 1,535
1–2	53.1	58.6	56.7	53.6		54.5
3–4	29.9	28.3	25.3	22.3		24.2
5+	16.9	13.1	18.0	24.2		21.2
**Birthweight, kg**	*n* = 161	*n* = 95	*n* = 309	*n* = 843	0.079	*n* = 1,408
<2.5	15.5	5.3	11.0	10.6		10.9
≥2.5	84.5	94.7	89.0	89.4		89.1
**Mother’s age at birth, y**	*n* = 177	*n* = 99	*n* = 344	*n* = 915	0.021	*n* = 1,535
<20	10.7	16.2	17.7	20.1		18.2
20–29	54.2	50.5	51.5	43.9		47.2
30+	35.0	33.3	30.8	36.0		34.5
**Mother’s HIV status**	*n* = 177	*n* = 99	*n* = 343	*n* = 914	<0.001	*n* = 1,533
Negative	22.6	43.4	25.7	66.5		50.8
Positive pregnancy	65.0	43.4	58.0	13.0		31.1
Positive since pregnancy	12.4	13.1	16.3	20.5		18.1
**Child hospitalizations (since birth)**	*n* = 177	*n* = 99	*n* = 344	*n* = 911	0.19	*n* = 1,531
0	84.2	82.8	85.8	88.4		86.9
1+	15.8	17.2	14.2	11.6		13.1
**Mother’s education**	*n* = 173	*n* = 94	*n* = 340	*n* = 905	0.0023	*n* = 1,512
None/primary	33.5	27.7	41.8	44.1		41.3
Some secondary or higher	66.5	72.3	58.2	55.9		58.7
**Maternal BMI**	*n* = 175	*n* = 99	*n* = 340	*n* = 904	0.011	*n* = 1,518
<18.5	7.4	3.0	7.9	4.2		5.3
18.5–24	33.1	24.2	29.7	24.9		26.9
25–29	20.6	33.3	24.7	27.3		26.4
30+	38.9	39.4	37.6	43.6		41.4
**Owns fridge**	*n* = 177	*n* = 99	*n* = 344	*n* = 915	0.18	*n* = 1,535
No	21.5	20.2	25.9	27.7		26.1
Yes	78.5	79.8	74.1	72.3		73.9

^a^Underfat defined using age- and sex-specific second percentile of body fat percentage from McCarthy reference.

**Abbreviations:** BMI, body mass index; NCD, noncommunicable disease

In multivariable analysis of overfat (including obese-fat; Tables 2, 3 and 4), adjusting for early life factors, normal birth weight children were almost 3 times more likely to be overfat than low birth weight children (95% confidence interval [CI] 1.08–7.97; *P* = 0.034). Independently, HIV-exposed children had almost 50% lowered odds of being overfat (95% CI [0.31–0.97]; *P* = 0.04). In the final model, adjusting for both early and current life factors, breastfeeding duration was strongly associated with being overfat: stopping breastfeeding between 6 and 11 or 12+ months decreased the odds of being overfat by approximately 50% compared with breastfeeding for less than 1 month or never breastfeeding (6–11 months 95% CI 0.21–0.91; *P* = 0.027). For normal birthweight and maternal HIV status, although the adjusted odds ratios (aORs) remained similar to the model adjusting for early life factors only, statistical significance was borderline after adjustment for current maternal BMI (*P* = 0.066 for normal weight; *P* = 0.22 for positive pregnancy). Children with an obese mother had 5 times the odds of being overfat compared with children with normal weight mothers (95% CI 2.47–10.20; *P* < 0.001). Based on predicted probabilities from the model estimates, a child breastfed for 12+ months had a 6% probability of being overfat compared with a 13% probability for children breastfed for <1 month. Including an interaction between breastfeeding duration and HIV-exposed and unexposed children did not significantly improve model fit (*χ*^2^(3) = 2.25; *P* = 0.52).

**Table 2:** Multivariable logistic regressions of overfat (including obese-fat) on maternal, child, and household early life and current factors, child ages 7 to 11 years (*n* = 1,364).

	Breastfeeding only			+ Early life factors			+ Current life factors		
	OR	95% CI	*p*-value	aOR	95% CI	*p*-value	aOR	95% CI	*p*-value
**Age stop any breastfeeding, mo**									
0	ref			ref			ref		
1–5	0.45	0.16–1.26	0.13	0.36	0.13–1.05	0.061	0.34	0.11–1.06	0.063
6–11	0.43	0.21–0.87	0.02	0.42	0.21–0.87	0.019	0.43	0.21–0.91	0.027
12+	0.57	0.33–1.00	0.051	0.44	0.23–0.82	0.0099	0.45	0.22–0.91	0.027

**Abbreviations:** aOR, adjusted odds ratio; CI, confidence interval; OR, odds ratio; ref, reference

**Table 3:** Multivariable logistic regressions of overfat (including obese-fat) on maternal, child, and household early life factors, child ages 7 to 11 years (*n* = 1,364).

	+ Early life factors			+ Current life factors		
	aOR	95% CI	*p*-value	aOR	95% CI	*p*-value
**Birth order**						
1–2	ref			ref		
3–4	1.09	0.62–1.93	0.75	1.11	0.63–1.95	0.72
5+	0.51	0.23–1.10	0.086	0.56	0.26–1.21	0.14
**Birth weight, kg**						
<2.5	ref			ref		
≥2.5	2.94	1.08–7.97	0.034	2.64	0.94–7.47	0.066
**Mother’s age (at birth), y**						
<20	ref			ref		
20–29	1.24	0.62–2.48	0.54	1.1	0.55–2.22	0.78
30+	2.06	0.88–4.82	0.096	1.71	0.73–4.01	0.22
**Mother’s HIV status**						
Negative	ref			ref		
Positive pregnancy	0.55	0.31–0.97	0.04	0.68	0.37–1.25	0.22
Positive since pregnancy	0.77	0.42, 1.41	0.4	1.05	0.57, 1.95	0.87

**Abbreviations:** aOR, adjusted odds ratio; CI, confidence interval; OR, odds ratio; ref, reference

**Table 4:** Multivariable logistic regressions of overfat (including obese-fat) on maternal, child, and household current life factors, child ages 7 to 11 years (*n* = 1,364).

	+ Current life factors		
	aOR	95% CI	*p*-value
**Stunting**			
< 2.5 kg	ref		
≥ 2.5 kg	0.95	[0.20, 4.40]	0.94
**Child hospitalizations (since birth)**			
0	ref		
1+	0.95	[0.49, 1.85]	0.88
**Mother’s education**			
None/primary	ref		
Some secondary or higher	1.36	[0.86, 2.16]	0.19
**Maternal current BMI**			
<18.5	1.07	[0.22, 5.18]	0.93
18.5–24	ref		
25–29	2.15	[0.94, 4.90]	0.068
30+	5.02	[2.47, 10.20]	<0.001
**Owns fridge**			
No	ref		
Yes	1.62	[0.90, 2.92]	0.11

**Abbreviations:** aOR, adjusted odds ratio; BMI, body mass index; CI, confidence interval; OR, odds ratio; ref, reference

Tables 5, 6 and 7 present the aORs from the regression of overweight (including obesity based on BMI). Breastfeeding duration of 6 to 11 or 12+ months lowered the odds of being overweight compared with breastfeeding for <1 month or never breastfeeding. For early life factors, normal birth weight children were over 2 times more likely to be overweight at ages 7 to 11 years compared with underweight children (95% CI 1.05–4.26; *P* = 0.036). In the final model including early and current life factors, the lowered odds of being overweight associated with increased breastfeeding duration remained, whereas the association with normal birth weight was in the same direction but borderline significant (*P* = 0.081) after adjusting for maternal BMI. Maternal BMI and education were the only current factors associated with overweight. Children with an obese mother had over 4 times the odds of being overweight compared with children with normal weight mothers (95% CI 2.65–7.09; *P* < 0.001). Children with a mother with some secondary education or higher had 1.7 times the odds of being overweight compared with children of mothers with lower education (95% CI 1.15–2.58; *P* = 0.0083). Including an interaction between breastfeeding duration and HIV-exposed and -unexposed children did not significantly improve model fit (*χ*^2^(3) = 2.16; *P* = 0.54).

**Table 5:** Multivariable logistic regressions of overweight (including obesity) on maternal, child, and household early life and current factors, child ages 7 to 11 years (*n* = 1,285).

	Breastfeeding only			+ Early life factors			+ Current life factors		
	OR	95% CI	*p*-value	aOR	95% CI	*p*-value	aOR	95% CI	*p*-value
**Age stop any breastfeeding, mo**									
0	ref			ref			ref		
1–5	0.48	0.23–1.02	0.055	0.42	0.19–0.91	0.029	0.4	0.18–0.90	0.027
6–11	0.48	0.28–0.82	0.0071	0.46	0.26–0.80	0.0058	0.46	0.26–0.82	0.0083
12+	0.51	0.33–0.79	0.0028	0.44	0.27–0.72	0.0012	0.46	0.26–0.79	0.005

**Abbreviations:** aOR, adjusted odds ratio; CI, confidence interval; OR, odds ratio; ref, reference

**Table 6:** Multivariable logistic regressions of overweight (including obesity) on maternal, child, and household early life factors, child ages 7 to 11 years (*n* = 1,285).

	+ Early life factors			+ Current life factors		
	aOR	95% CI	*p*-value	aOR	95% CI	*p*-value
**Birth order**						
1–2	ref			ref		
3–4	0.85	0.55–1.32	0.47	0.9	0.58–1.41	0.65
5+	0.74	0.42–1.31	0.3	0.92	0.51–1.68	0.8
**Birthweight, kg**						
<2.5	ref			ref		
≥2.5	2.12	1.05–4.26	0.036	1.92	0.92–3.98	0.081
**Mother’s age (at birth), y**						
<20	ref			ref		
20–29	1.11	0.68–1.80	0.67	1	0.61–1.64	1
30+	1.26	0.67–2.36	0.47	1.1	0.58–2.08	0.77
**Mother’s HIV status**						
Negative	ref			ref		
Positive pregnancy	0.77	0.51–1.16	0.21	0.96	0.62–1.51	0.87
Positive since pregnancy	0.78	0.49–1.22	0.28	1.04	0.65–1.66	0.88

**Abbreviations:** aOR, adjusted odds ratio; CI, confidence interval; OR, odds ratio; ref, reference

**Table 7:** Multivariable logistic regressions of overweight (including obesity) on maternal, child, and household current life factors, child ages 7 to 11 years (*n* = 1,285).

	+ Current life factors		
	aOR	95% CI	*p*-value
**Child hospitalizations (since birth)**			
0	ref		
1+	0.78	0.46–1.31	0.34
**Mother’s education**			
None/primary	ref		
Some secondary or higher	1.72	1.15–2.58	0.0083
**Maternal BMI**			
<18.5	Omitted^1^		
18.5–24	ref		
25–29	2.03	1.17–3.53	0.012
30+	4.33	2.65–7.09	<0.001
**Owns fridge**			
No	ref		
Yes	1.51	0.98–2.33	0.065

^1^Omitted due to small sample size.

**Abbreviations:** aOR, adjusted odds ratio; BMI, body mass index; CI, confidence interval; OR, odds ratio; ref, reference

Tables 8, 9 and 10 show the associations of prehypertension (including hypertension) and breastfeeding, early life, and current factors. The inverse association between prehypertension at 7 to 11 years and breastfeeding duration did not reach statistical significance. None of the other factors examined, including maternal HIV status, were statistically significantly associated with prehypertension. An interaction between breastfeeding duration and HIV-exposed and -unexposed children did not significantly improve model fit (*χ*^2^(3) = 2.68; *P* = 0.44).

**Table 8:** Multivariable logistic regressions of prehypertension (including hypertension) on maternal, child, and household early life and current factors, child ages 7 to 11 years (*n* = 1,364).

	Breastfeeding only			+ Early life factors			+ Current life factors		
	OR	95% CI	*p*-value	aOR	95% CI	*p*-value	aOR	95% CI	*p*-value
**Age stop any breastfeeding, mo**									
0	ref			ref			ref		
1–5	0.52	0.20–1.34	0.17	0.53	0.20–1.40	0.2	0.52	0.20–1.40	0.2
6–11	0.69	0.37–1.28	0.24	0.71	0.38–1.34	0.3	0.72	0.38–1.37	0.32
12+	0.69	0.41–1.18	0.18	0.68	0.37–1.25	0.21	0.68	0.36–1.27	0.22

**Abbreviations:** aOR, adjusted odds ratio; CI, confidence interval; OR, odds ratio; ref, reference

**Table 9:** Multivariable logistic regressions of prehypertension (including hypertension) on maternal, child, and household early life factors, child ages 7 to 11 years (*n* = 1,364).

	+ Early life factors			+ Current life factors		
	aOR	95% CI	*p*-value	aOR	95% CI	*p*-value
**Birth order**						
1–2	ref			ref		
3–4	1.17	0.72–1.90	0.52	1.16	0.71–1.88	0.55
5+	1.03	0.56–1.89	0.92	1.01	0.54–1.89	0.98
**Birthweight, kg**						
<2.5	ref			ref		
≥2.5	0.67	0.39–1.16	0.15	0.66	0.38–1.13	0.13
**Mother’s age (at birth), y**						
<20	ref			ref		
20–29	1.44	0.77–2.70	0.25	1.45	0.77–2.72	0.25
30+	1.65	0.77–3.53	0.2	1.61	0.74–3.47	0.23
**Mother’s HIV status**						
Negative	ref			ref		
Positive pregnancy	0.83	0.50–1.39	0.48	0.83	0.49–1.41	0.5
Positive since pregnancy	0.72	0.41–1.28	0.27	0.74	0.41–1.33	0.32

**Abbreviations:** aOR, adjusted odds ratio; CI, confidence interval; OR, odds ratio; ref, reference

**Table 10:** Multivariable logistic regressions of prehypertension (including hypertension) on maternal, child, and household current life factors, child ages 7 to 11 years (*n* = 1,364).

	+ Current life factors		
	aOR	95% CI	*p*-value
**Child hospitalizations (since birth)**			
0	ref		
1+	0.95	0.54–1.66	0.86
**Mother’s education**			
None/primary	ref		
Some secondary or higher	0.92	0.60–1.42	0.71
**Maternal BMI**			
<18.5	0.77	0.29–2.08	0.61
18.5–24	ref		
25–29	1.09	0.64–1.84	0.76
30+	1.02	0.63–1.66	0.92
**Owns fridge**			
No	ref		
Yes	1.33	0.83–2.14	0.24

**Abbreviations:** aOR, adjusted odds ratio; BMI, boby mass index; CI, confidence interval; OR, odds ratio; ref, reference

To assess the accuracy of breastfeeding recall, S1 Table presents a comparison between breastfeeding duration based on recall and using prospectively collected breastfeeding data for the VTS subsample only. Recall was generally accurate for those who breastfed for <1 month (82% of those who were prospectively measured at <1 month correctly recalled breastfeeding for <1 month), 1 to 5 months (73%), and 12+ months (80%). However, differentiation for those who breastfed 6 to 11 months was lower, with recall roughly split between 6 and 11 months (39%) and 12+ months (46%). In the final model for each outcome, the associations with breastfeeding duration based on prospective feeding data in the VTS subsample were similar to those based on breastfeeding recall in the final sample (S2 Table) but were not statistically significant, which is likely due to reduced sample size.

Finally, to assess the sensitivity of our results to dichotomizing our outcome variables, S3 Table and S4 Table present results from the quantile regressions. These models show similar results to the logistic regressions for overfat and overweight. The SBP and DBP models showed a protective effect for breastfeeding for 12+ months. For instance, breastfeeding for 12+ months reduced the DBP z score of children with DBP values on the 91st percentile by −0.3 on average compared with those children breastfed for less than 1 month (95% CI −0.52 to −0.06; *P* = 0.013). For the underfat model (S4 Table), breastfeeding for 12+ months reduced the body fat percent of children on the 35th percentile by −0.59 on average (95% CI −1.14 to −0.04; *P* = 0.036). Similarly, for the underweight model (S4 Table), breastfeeding for 12+ months reduced the BMI z score of children on the fourth percentile by −0.57 on average (95% CI −0.97 to −0.18; *P* = 0.0044).

## Discussion

To our knowledge, this is the first study examining breastfeeding, obesity, overweight, and BP in HIV-exposed and unexposed school-aged children in Africa. Seven percent of 7 to 11 year-old children were overfat, 13% overweight, and 9% prehypertensive in this rural, high HIV prevalence setting in South Africa. Our finding that sustained breastfeeding beyond 6 months is associated with approximately halved odds of being overfat and overweight at ages 7 to 11 years, irrespective of maternal HIV status and allowing for early and current life factors, is important given the increasing obesity epidemic, particularly in resource-poor settings, including those with high HIV prevalence [23]. Our finding reinforces the current WHO guidelines on sustained breastfeeding for HIV-infected women on ART [65], bringing the breastfeeding guidelines in line with those for uninfected women. Although we are unaware of studies examining overfatness in South African children, results from the PROMISE-EBF trial in South Africa showed that early nonbreastfeeding was associated with obesity at 2 years but this study excluded children of HIV-positive mothers [15]. Our findings are consistent with data from large cohorts in high-income countries [8,13,17,18] and with a meta-analysis [11] estimating a 4% decreased risk of being overweight for each month of breastfeeding up to 9 months of age, which may be important at a population level. In an updated meta-analysis [19] of breastfeeding and later outcomes over a wide age range, breastfed children were reported to be less likely to be overweight and obese (pooled OR = 0.74; 95% CI 0.7–0.78). Among the high-quality studies included, breastfeeding was associated with an estimated 13% reduction in being overweight or obese in childhood [19].

There are many plausible theories as to why early breastfeeding may protect against later obesity. These include the optimal nutrition provided by breastmilk, including lower protein and higher fat content compared with formula milks [66], data suggesting breastfed infants are more able to self-regulate the amount they consume [67], increased gut Bifidobacteria thought to protect against childhood obesity [68], and new data suggesting an interaction between breastmilk and the FTO allele (fat mass and obesity gene) involved in the hypothalamic regulation of appetite and energy expenditure and associated with increased BMI in adolescence [11,69].

An important strength of our study was the inclusion of children of both HIV-positive and -negative mothers. In the absence of maternal HIV treatment, children born to HIV-infected women have lower birth weights [25–28] and are more likely to be small for gestational age than children of HIV-uninfected women [29]. Large cohorts, including our South African VTS cohort, have shown normal subsequent growth of HIV-exposed compared to HIV-unexposed children [30–32]. In our VTS cohort, we postulated that exclusive breastfeeding in the first 6 months, with benefits of optimal nutrition and protection from diarrhea and lower respiratory tract infections [70], compensated for the vulnerabilities of being born to an HIV-infected mother. In the current cohort study, we also found no differences between HIV-exposed and -unexposed children in any of the outcomes examined. Much of the existing evidence has been limited by not including follow-up maternal HIV testing, not including HIV-unexposed comparison groups, and in contemporary studies the inability to distinguish between the effects of HIV and ART exposures [71]. A small study in Zambia also found no significant differences between HIV-exposed and -unexposed children in subsequent growth and chronic disease markers after adjusting for SES [72].

We report an association between longer breastfeeding (i.e., 12+ months) and being underfat and underweight in midchildhood. Beyond 6 months of age, breastmilk alone is no longer sufficient to meet the infant’s energy requirements, and children require good quality complementary feeds. Breastmilk provides around half the energy needs between 6 and 12 months of age, and around one-third between 12 and 24 months [73]. It is possible that some of these children in our study were taking large quantities of breastmilk and thus suppressing their appetites for solid foods. It is also possible that mothers who continued to breastfeed did not have the resources to provide adequate complementary food. It is estimated that fewer than one-third of children aged 6 to 23 months in developing countries meet the minimum criteria for diversity and quality of complementary foods in their diets [74].

In line with prior studies [75–77], we showed a strong association between maternal current obesity and children’s risk of being overfat or obese. The pathways to obesity are complex, and there is increasing interest in the intrauterine environment and the fetal response to variations in this milieu at influential points of developmental plasticity and the effect on long-term outcomes [78]. In terms of the life course, obese children are on a trajectory to become obese adults and for female children to become overfat mothers [79]. Using current maternal BMI, we show that maternal obesity is associated with approximately 5 times the odds of their children being overfat and 4 times the odds of being overweight.

Although 9% of children were already in the prehypertension category (similar to an estimate of 8% for South Africa from a recent meta-analysis [42]), we found no associations with the factors we examined, although the ORs for breastfeeding were in the same direction as described for overweight and overfat, and the continuous models showed a protective effect at 12+ months for SBP and DBP. Our findings align with data from the Pelotas cohort in Brazil that showed no association between breastfeeding and BP, but not with data from the United Kingdom Avon Longitudinal Study of Parents and Children (ALSPAC) cohort which showed a 0.2 mmHg reduction in SBP for each 3 months of breastfeeding [80]. Brion and colleagues [80] have compared these cohorts, noting that in ALSPAC, breastfeeding was strongly associated with SES, which was not the case in the Brazilian Pelotas cohort, and that residual confounding could account for these differences. In our study, we used fridge ownership as a proxy for SES, which we identified as important in principal component analysis; however, given the study design, we are unable to fully account for residual confounding. Also, although a lack of SES confounding may partly explain our finding of a lack of association with BP, it does not explain the fact that our results showed an association between breastfeeding and overweight and overfat. In a meta-analysis examining the effect of breastfeeding on BP later in life, Owen and colleagues demonstrated only a small effect with limited public health importance. They cautioned that although the small studies in the analysis reported an association between breastfeeding and later BP, those studies with more than 1,000 participants showed little or no difference, suggesting that such findings could be due to publication bias [81].

The study limitations include that breastfeeding data were based on maternal recall 7 to 11 years after delivery. The literature suggests that recall on any breastfeeding, including in excess of 10 years, is accurate to within 1 month [82,83]. In our subanalysis of VTS children with prospective feeding data available, we found relatively accurate recall for less than 1, 1 to 5, and 12 or more months duration but lower accuracy for 6 to 11 months. This may at least partly explain why our estimates for the longer breastfeeding durations are similar (i.e., at 6–11 and 12+ months; although associations were similar in the VTS subsample) and limits our ability to differentiate on the effects of longer-term breastfeeding on children’s odds of being overweight or overfat. Maternal recall (and associated heaping of data) also limits our ability to operationalize breastfeeding in alternate ways such as continuously. We are unable to examine the age at which (and type of) other foods or liquids were introduced into the diets of children, which could influence their BMI [84] and body fat. Further, we do not have prepregnancy BMI data for the mothers; the link between maternal and child obesity could be due to the shared environment. However, in a subcomparison of VTS mothers, 87% who were obese 6 months postnatal were also obese at the follow-up when the children were in primary school. In the absence of other available reference data, we used McCarthy body fat reference curves, which is a limitation because they are derived for Caucasian children only. We also do not have information on other confounders such as diet or physical activity; however, a study from the same setting showed that few children met international physical activity recommendations [85].

The strengths include using one of the largest cohorts able to compare these outcomes in HIV-exposed and -unexposed children in Africa. Few women in the cohort did not breastfeed at all. We have measurement of overfatness in addition to BMI on all children using measures previously used in this study population and data on a number of factors likely to affect the outcomes, including SES.

## Conclusion

To our knowledge, this is the first study examining breastfeeding and childhood obesity in an African setting with high HIV prevalence. HIV has impacted breastfeeding guidelines over several decades [61,65,86–88], with confused and mixed messages about infant feeding and erosion of breastfeeding practices [20]. With the advent of ART for life, breastfeeding is recommended in many settings for HIV-positive women for at least 12 months and up to 24 months or longer [65]. Although evidence generally supports a protective association between breastfeeding and later overweight in childhood [19], it is important to examine this association in HIV-prevalent settings, particularly because breastfeeding rates may be less than optimum. We confirm and quantify the association, finding that breastfeeding for at least 6 months was associated with half the odds of being overfat and overweight in children aged 7 to 11 years; the association with BP, although in the same direction, did not reach statistical significance. Children of HIV-positive and HIV-negative mothers had similar outcomes in terms of their body fat, BMI, and BP. Breastfeeding has benefits that extend into midchildhood and should be highlighted, for both HIV-exposed and -unexposed children, as one of the strategies to tackle adult obesity and the associated public health consequences of ill health, including diabetes, hypertension, and cancer.

## Supporting information

S1 STROBE Checklist Checklist of items that should be included in reports of observational studies. STROBE, Strengthening the Reporting of Observational Studies in Epidemiology.
(DOCX)

S1 Table Comparison of breastfeeding duration between maternal recall and prospective feeding information, VTS subsample only (*n* = 905). VTS, Vertical Transmission Study.
(DOCX)

S2 Table Multivariable logistic regressions of overfat, overweight, and prehypertension on maternal, child, and household early life and current factors, child ages 7 to 11 years, VTS subsample only. Breastfeeding duration based on prospective feeding data. VTS, Vertical Transmission Study.
(DOCX)

S3 Table Multivariable quantile regressions for sample percentiles at clinical thresholds of overfat (body fat percent), overweight (BMI z score), and prehypertension (SBP and DBP z scores) on maternal, child, and household early life and current factors, child ages 7 to 11 years. BMI, body mass index; DBP, diastolic blood pressure; SBP, systolic blood pressure.
(DOCX)

S4 Table Multivariable quantile regressions for sample percentiles at clinical thresholds of underfat (body fat percent) and underweight (BMI z score) on maternal, child, and household early life and current factors, child ages 7 to 11 years. BMI, body mass index.
(DOCX)

## Abbreviations

AHRI: Africa Health Research Institute
ALSPAC: Avon Longitudinal Study of Parents and Children
aOR: adjusted odds ratio
ART: antiretroviral treatment
BMI: body mass index
BP: blood pressure
CI: confidence interval
DBP: diastolic blood pressure
DSS: demographic surveillance system
FTO: fat mass and obesity gene
IOTF: International Obesity Task Forc
LMIC: low- and middle-income countries
NCD: noncommunicable disease
OR: odds ratio
PMTCT: prevention of mother-to-child transmission
SBP: systolic blood pressure
SES: socioeconomic status
STROBE: Strengthening the Reporting of Observational Studies in Epidemiology
VTS: Vertical Transmission Study
WHO: World Health Organization
