# Supplementary material for: Breastfeeding, HIV exposure, childhood obesity, and prehypertension: A South African cohort study
Source: PLoS Med. 2019 Aug 27;16(8):e1002889. doi: 10.1371/journal.pmed.1002889 (PMC6711496; doi:10.1371/journal.pmed.1002889)
Supplement: S1 STROBE Checklist — STROBE, Strengthening the Reporting of Observational Studies in Epidemiology. (DOCX) [file pmed.1002889.s001.docx]

**S1 STROBE Checklist**. Checklist of items that should be included in reports of observational studies

|  | Item No. | Recommendation | Page  No. | Relevant text from manuscript |
| --- | --- | --- | --- | --- |
| **Title and abstract** | 1 | (*a*) Indicate the study’s design with a commonly used term in the title or the abstract | Title and abstract | Title indicates the study design “a cohort study” |
|  |  | (*b*) Provide in the abstract an informative and balanced summary of what was done and what was found | Abstract | Abstract provides an informative and balanced summary. |
| Introduction | | | |  |
| Background/rationale | 2 | Explain the scientific background and rationale for the investigation being reported | Introduction  Paragraphs 1-4 | Paragraph 1 outlines the importance of NCDs and their link to early life exposures, including optimal nutrition for the child in the first 1000 days of life.  Paragraph 2 highlights the limitations of the existing evidence, including: lack of evidence from low-resource settings; small sample sizes; and lack of adjustment for confounding factors. It also highlights the importance of additional studies in HIV-endemic regions.  Paragraph 3 summarizes the links between breastfeeding, HIV exposure and obesity.  Paragraph 4 outlines the cohort under investigation in this manuscript and the importance of the study setting. |
| Objectives | 3 | State specific objectives, including any prespecified hypotheses | Introduction  Paragraph 4 lines 1-3  Paragraph 5 lines 3-5 | Paragraph 4 lines 1-3 clearly states the objectives of the investigation. Paragraph 5 outlines the aims on lines 1-3.  Paragraph 5 lines 3-5 includes our hypothesis. |
| Methods | | | |  |
| Study design | 4 | Present key elements of study design early in the paper | Methods  Paragraph 1 | Paragraph 1 outlines the study design and key characteristics of the sample. |
| Setting | 5 | Describe the setting, locations, and relevant dates, including periods of recruitment, exposure, follow-up, and data collection | Methods  Paragraphs 1-2 | Paragraph 1 provides information on the study setting and relevant dates of the current study along with dates for the original VTS study.  Paragraph 2, sub section heading “Data collection” we provide dates for recruitment of the current follow-up and a clear description of the data collection process. |
| Participants | 6 | (*a*) *Cohort study*—Give the eligibility criteria, and the sources and methods of selection of participants. Describe methods of follow-up  *Case-control study*—Give the eligibility criteria, and the sources and methods of case ascertainment and control selection. Give the rationale for the choice of cases and controls  *Cross-sectional study*—Give the eligibility criteria, and the sources and methods of selection of participants | Methods  Paragraph 1  Data collection  Figure 1 | Eligibility: Paragraph 1 describes the eligibility of the participants.  Sources and methods of selection: Paragraph 1 describes the sources of both the VTS and DSS children included in the sample.  Methods of follow-up: The sub heading “Data collection” describes the method of follow-up in detail.  We provide a participant flowchart in Figure 1 that illustrates the above points as well. |
|  |  | (*b*) *Cohort study*—For matched studies, give matching criteria and number of exposed and unexposed  *Case-control study*—For matched studies, give matching criteria and the number of controls per case |  |  |
| Variables | 7 | Clearly define all outcomes, exposures, predictors, potential confounders, and effect modifiers. Give diagnostic criteria, if applicable | Methods  Child outcome measures  Data collection  Statistical analyses | The sub heading “Child outcome measures” clearly defines the three outcomes and how they were measured.  The sub heading “Data collection” defines the data collection methods.  The sub heading “Statistical analyses” defines the predictors and how they were categorized in the analyses. |
| Data sources/ measurement | 8* | For each variable of interest, give sources of data and details of methods of assessment (measurement). Describe comparability of assessment methods if there is more than one group | Methods  Data collection  Child outcome measures | We have described the data sources and methods in the sub headings “Data collection” and “Child outcome measures”. |
| Bias | 9 | Describe any efforts to address potential sources of bias | Methods  Statistical analyses paragraph 3 | Paragraph 3 in sub heading “Statistical analyses” addresses potential biases between breastfeeding duration based on recall compared to prospective data collection in the VTS subsample. |
| Study size | 10 | Explain how the study size was arrived at | Figure 1 | Figure 1 describes how the final estimation sample was arrived at for these analyses. |

| Quantitative variables | 11 | Explain how quantitative variables were handled in the analyses. If applicable, describe which groupings were chosen and why | Methods  Statistical analyses paragraphs 1-2 | In sub heading “Statistical analyses” paragraphs 1-2 we explain how each of the quantitative variables was addressed in the analysis. |
| --- | --- | --- | --- | --- |
| Statistical methods | 12 | (*a*) Describe all statistical methods, including those used to control for confounding | Methods  Statistical analyses | In the sub heading “Statistical analyses” we describe the following:   1. Statistical methods 2. Testing for interactions 3. How missing data were treated in the analyses 4. Loss to follow-up is presented in Figure 1 5. Sensitivity analyses are listed in paragraphs 3 and 4 |
|  |  | (*b*) Describe any methods used to examine subgroups and interactions |  |  |
|  |  | (*c*) Explain how missing data were addressed |  |  |
|  |  | (*d*) *Cohort study*—If applicable, explain how loss to follow-up was addressed  *Case-control study*—If applicable, explain how matching of cases and controls was addressed  *Cross-sectional study*—If applicable, describe analytical methods taking account of sampling strategy |  |  |
|  |  | (*e*) Describe any sensitivity analyses |  |  |
| Results | | | | |
| Participants | 13* | (a) Report numbers of individuals at each stage of study—eg numbers potentially eligible, examined for eligibility, confirmed eligible, included in the study, completing follow-up, and analysed | Results  Paragraph 1  Figure 1 | Figure 1 describes the number of individuals at each stage of the study, reasons for non-participation, and uses a flow diagram to illustrate these numbers. See also paragraph 1. |
|  |  | (b) Give reasons for non-participation at each stage |  |  |
|  |  | (c) Consider use of a flow diagram |  |  |
| Descriptive data | 14* | (a) Give characteristics of study participants (eg demographic, clinical, social) and information on exposures and potential confounders | Results  Table 1 | Table 1 provides descriptions of characteristics of the participants.  Table 1 also indicates the number of participants with missing data, by variable. |
|  |  | (b) Indicate number of participants with missing data for each variable of interest |  |  |
|  |  | (c) *Cohort study*—Summarise follow-up time (eg, average and total amount) |  |  |
| Outcome data | 15* | *Cohort study*—Report numbers of outcome events or summary measures over time | Results  Paragraph 2 lines 6-10  Table 1 | Table 1 shows data for each outcome by breastfeeding duration and overall.  Paragraph 2 lines 6-10 also outlines outcomes overall. |
|  |  | *Case-control study—*Report numbers in each exposure category, or summary measures of exposure |  |  |
|  |  | *Cross-sectional study—*Report numbers of outcome events or summary measures |  |  |
| Main results | 16 | (*a*) Give unadjusted estimates and, if applicable, confounder-adjusted estimates and their precision (eg, 95% confidence interval). Make clear which confounders were adjusted for and why they were included | Results  Tables 2-4  Methods  Child outcome measures  Statistical analyses | Tables 2-4 give unadjusted estimates for breastfeeding duration for each of the three outcomes. Subsequent models then adjust for early life and then early life and current life factors. Each table also reports the boundaries for each of the predictors.  Section head “Methods” sub head “Child outcome measures” details how boundaries were defined for the outcome variables. Sub head “Statistical analyses” details how boundaries were defined for the predictor variables. |
|  |  | (*b*) Report category boundaries when continuous variables were categorized |  |  |
|  |  | (*c*) If relevant, consider translating estimates of relative risk into absolute risk for a meaningful time period |  |  |

| Other analyses | 17 | Report other analyses done—eg analyses of subgroups and interactions, and sensitivity analyses | Methods  Statistical analyses paragraphs 3-4  Results paragraphs 6-7 | In section head “Methods” sub head “Statistical analyses” paragraphs 3-4 outline additional analyses conducted.  In section head “Results” paragraphs 6-7 we report on the results of the additional analyses conducted. |
| --- | --- | --- | --- | --- |
| Discussion | | | | |
| Key results | 18 | Summarise key results with reference to study objectives | Discussion  Paragraphs 1 and 3-6 | Paragraph 1 summarizes the main results of the study. Paragraphs 3-6 compare our results to those from other studies. |
| Limitations | 19 | Discuss limitations of the study, taking into account sources of potential bias or imprecision. Discuss both direction and magnitude of any potential bias | Discussion  Paragraph 7 | Paragraph 7 outlines the limitations of the study. |
| Interpretation | 20 | Give a cautious overall interpretation of results considering objectives, limitations, multiplicity of analyses, results from similar studies, and other relevant evidence | Discussion  Paragraphs 3-6 and 9 | Paragraphs 3-6 compare our results to those from other studies and potential explanations. Paragraph 9 gives the overall interpretation of the study’s results. |
| Generalisability | 21 | Discuss the generalisability (external validity) of the study results | Discussion  Paragraphs 1 and 3-6 | In paragraph 1 we compare our results with studies in South Africa, other cohorts, and a meta-analysis of breastfeeding and later outcomes. In paragraph 3 we discuss HIV exposure relating to our results and those of other studies. Paragraph 4 discusses findings related to underfat and underweight. Paragraph 5 shows that our findings on obesity align with prior studies. Paragraph 6 discusses similarities and differences between our results and two other studies on prehypertension. |
| Other information | |  | | |
| Funding | 22 | Give the source of funding and the role of the funders for the present study and, if applicable, for the original study on which the present article is based | Funding section | We identify the funder in the funding section of the manuscript. |

*Give information separately for cases and controls in case-control studies and, if applicable, for exposed and unexposed groups in cohort and cross-sectional studies.

**Note:** An Explanation and Elaboration article discusses each checklist item and gives methodological background and published examples of transparent reporting. The STROBE checklist is best used in conjunction with this article (freely available on the Web sites of PLoS Medicine at http://www.plosmedicine.org/, Annals of Internal Medicine at http://www.annals.org/, and Epidemiology at http://www.epidem.com/). Information on the STROBE Initiative is available at www.strobe-statement.org.
