# Supplementary material for: Breastfeeding, HIV exposure, childhood obesity, and prehypertension: A South African cohort study
Source: PLoS Med. 2019 Aug 27;16(8):e1002889. doi: 10.1371/journal.pmed.1002889 (PMC6711496; doi:10.1371/journal.pmed.1002889)
Supplement: S1 Table — VTS, Vertical Transmission Study. (DOCX) [file pmed.1002889.s002.docx]

**S1 Table.** Comparison of breastfeeding duration between maternal recall and prospective feeding information, VTS subsample only (n=905).

|  | **Measured^1^** | | | | | | | | | |
| --- | --- | --- | --- | --- | --- | --- | --- | --- | --- | --- |
|  | **<1 month** | | **1-5 months** | | **6-11 months** | | **12+ months** | | |  |
| **Recall** | **N** | **%** | **N** | **%** | **N** | **%** | | **N** | **%** | |
| <1 month | 37 | 82.2 | 1 | 9.1 | 39 | 8.8 | | 7 | 1.7 | |
| 1-5 months | 2 | 4.4 | 8 | 72.7 | 31 | 7.0 | | 2 | 0.5 | |
| 6-11 months | 3 | 6.7 | 1 | 9.1 | 171 | 38.6 | | 72 | 17.7 | |
| 12+ months | 3 | 6.7 | 1 | 9.1 | 202 | 45.6 | | 325 | 80.0 | |
| **Total** | 45 | 100.0 | 11 | 100.0 | 443 | 100.0 | | 406 | 100.0 | |

^1^ In VTS feeding data were collected by field monitors who visited the mothers every week and documented all feeds (milk and solids) and other fluids for each day of the preceding week. Mothers kept food-intake diaries for use during the field monitor interview to corroborate the verbal report.
