# Supplementary material for: Breastfeeding, HIV exposure, childhood obesity, and prehypertension: A South African cohort study
Source: PLoS Med. 2019 Aug 27;16(8):e1002889. doi: 10.1371/journal.pmed.1002889 (PMC6711496; doi:10.1371/journal.pmed.1002889)
Supplement: S2 Table — Breastfeeding duration based on prospective feeding data. VTS, Vertical Transmission Study. (DOCX) [file pmed.1002889.s003.docx]

**S2 Table.** Multivariable logistic regressions of overfat, overweight and prehypertension on maternal, child, and household early life and current factors, child ages 7-11 years, VTS sub-sample only. Breastfeeding duration based on prospective feeding data.

|  | **Overfat (n=825)** | | | **Overweight (n=787)** | | | **Prehypertension (n=836)** | | |
| --- | --- | --- | --- | --- | --- | --- | --- | --- | --- |
|  | **aOR** | **95% CI** | **p-value** | **aOR** | **95% CI** | **p-value** | **aOR** | **95% CI** | **p-value** |
| Age stop any breastfeeding, mo |  |  |  |  |  |  |  |  |  |
| 0 | ref |  |  | ref |  |  | ref |  |  |
| 1-5 | Omitted^1^ |  |  | 0.3 | [0.03, 3.28] | 0.33 | 1.16 | [0.19, 7.08] | 0.88 |
| 6-11 | 0.49 | [0.17, 1.47] | 0.21 | 0.44 | [0.18, 1.03] | 0.058 | 0.63 | [0.24, 1.64] | 0.34 |
| 12+ | 0.4 | [0.12, 1.31] | 0.13 | 0.6 | [0.25, 1.42] | 0.24 | 0.55 | [0.20, 1.50] | 0.25 |
| **Early life factors** |  |  |  |  |  |  |  |  |  |
| Birth order |  |  |  |  |  |  |  |  |  |
| 1-2 | ref |  |  | ref |  |  | ref |  |  |
| 3-4 | 1.01 | [0.41, 2.47] | 0.98 | 0.73 | [0.40, 1.33] | 0.3 | 1.53 | [0.85, 2.72] | 0.15 |
| 5+ | 0.64 | [0.17, 2.36] | 0.5 | 0.86 | [0.37, 2.00] | 0.73 | 0.76 | [0.35, 1.64] | 0.48 |
| Birthweight, kg |  |  |  |  |  |  |  |  |  |
| <2.5 | ref |  |  | ref |  |  | ref |  |  |
| ≥2.5 | 5.9 | [0.76, 46.04] | 0.09 | 2 | [0.75, 5.33] | 0.16 | 0.7 | [0.34, 1.46] | 0.35 |
| Mother’s age (at birth), y |  |  |  |  |  |  |  |  |  |
| <20 | ref |  |  | ref |  |  | ref |  |  |
| 20-29 | 0.95 | [0.38, 2.38] | 0.92 | 0.96 | [0.53, 1.73] | 0.88 | 1.62 | [0.73, 3.58] | 0.23 |
| 30+ | 1.64 | [0.45, 5.95] | 0.45 | 1.17 | [0.52, 2.67] | 0.7 | 2.54 | [1.01, 6.39] | 0.048 |
| Mother’s HIV status |  |  |  |  |  |  |  |  |  |
| Negative | ref |  |  | ref |  |  | ref |  |  |
| Positive pregnancy | 1.4 | [0.70, 2.78] | 0.34 | 1.28 | [0.79, 2.07] | 0.32 | 0.72 | [0.41, 1.25] | 0.24 |
| Positive since pregnancy | 1.38 | [0.55, 3.46] | 0.5 | 1.07 | [0.57, 2.02] | 0.83 | 0.65 | [0.31, 1.36] | 0.25 |
| **Current life factors** |  |  |  |  |  |  |  |  |  |
| Child hospitalizations (since birth) |  |  |  |  |  |  |  |  |  |
| 0 | ref |  |  | ref |  |  | ref |  |  |
| 1+ | 1.17 | [0.48, 2.86] | 0.73 | 0.96 | [0.49, 1.89] | 0.91 | 0.84 | [0.40, 1.76] | 0.64 |
| Mother’s education |  |  |  |  |  |  |  |  |  |
| None/primary | ref |  |  | ref |  |  | ref |  |  |
| Some secondary or higher | 2.08 | [1.01, 4.30] | 0.048 | 2.51 | [1.45, 4.36] | 0.0011 | 0.96 | [0.59, 1.58] | 0.88 |
| Maternal current BMI |  |  |  |  |  |  |  |  |  |
| <18.5 | 0.93 | [0.10, 8.40] | 0.95 | Omitted^1^ |  |  | 0.44 | [0.10, 1.97] | 0.28 |
| 18.5-24 | ref |  |  | ref |  |  | ref |  |  |
| 25-29 | 2.27 | [0.75, 6.83] | 0.15 | 2.42 | [1.21, 4.85] | 0.012 | 0.93 | [0.49, 1.77] | 0.82 |
| 30+ | 4.39 | [1.64, 11.75] | 0.0032 | 4.39 | [2.29, 8.39] | <0.001 | 0.76 | [0.42, 1.37] | 0.36 |
| Owns fridge |  |  |  |  |  |  |  |  |  |
| No | ref |  |  | ref |  |  | ref |  |  |
| Yes | 2.01 | [0.81, 4.97] | 0.13 | 1.47 | [0.84, 2.55] | 0.17 | 1.34 | [0.75, 2.41] | 0.32 |
| Stunting |  |  |  |  |  |  |  |  |  |
| < 2.5 kg | ref |  |  |  |  |  |  |  |  |
| ≥ 2.5 kg | 2.5 | [0.49, 12.62] | 0.27 |  |  |  |  |  |  |

OR indicates odds ratio; aOR indicates adjusted odds ratio; CI indicates confidence interval.

^1^ Omitted due to small sample size.
