# Supplementary material for: Breastfeeding, HIV exposure, childhood obesity, and prehypertension: A South African cohort study
Source: PLoS Med. 2019 Aug 27;16(8):e1002889. doi: 10.1371/journal.pmed.1002889 (PMC6711496; doi:10.1371/journal.pmed.1002889)
Supplement: S3 Table — BMI, body mass index; DBP, diastolic blood pressure; SBP, systolic blood pressure. (DOCX) [file pmed.1002889.s004.docx]

**S3 Table.** Multivariable quantile regressions for sample percentiles at clinical thresholds of overfat (body fat percent), overweight (BMI z-score) and prehypertension (SBP and DBP z-scores) on maternal, child, and household early life and current factors, child ages 7-11 years.

|  | **Body fat % (n=1364)^1^** | | | **BMI z-score (n=1361)** | | | **SBP (n=1364)** | | | **DBP (n=1364)** | | |
| --- | --- | --- | --- | --- | --- | --- | --- | --- | --- | --- | --- | --- |
|  | **B** | **95% CI** | **p-value** | **B** | **95% CI** | **p-value** | **B** | **95% CI** | **p-value** | **B** | **95% CI** | **p-value** |
| Age stop any breastfeeding, mo |  |  |  |  |  |  |  |  |  |  |  |  |
| 0 | ref |  |  | ref |  |  | ref |  |  |  |  |  |
| 1-5 | -6.25 | [-10.39, -2.11] | 0.0031 | -0.59 | [-0.96, -0.22] | 0.002 | -0.37 | [-0.70, -0.04] | 0.027 | -0.17 | [-0.44, 0.10] | 0.21 |
| 6-11 | -4.29 | [-8.23, -0.35] | 0.033 | -0.47 | [-0.86, -0.08] | 0.018 | -0.2 | [-0.44, 0.05] | 0.12 | 0.04 | [-0.22, 0.30] | 0.74 |
| 12+ | -3.75 | [-7.63, 0.13] | 0.058 | -0.47 | [-0.85, -0.09] | 0.015 | -0.24 | [-0.48, -0.00] | 0.045 | -0.29 | [-0.52, -0.06] | 0.013 |
| **Early life factors** |  |  |  |  |  |  |  |  |  |  |  |  |
| Birth order |  |  |  |  |  |  |  |  |  |  |  |  |
| 1-2 | ref |  |  | ref |  |  | ref |  |  |  |  |  |
| 3-4 | -0.86 | [-2.99, 1.27] | 0.43 | 0.07 | [-0.15, 0.29] | 0.54 | 0.21 | [-0.07, 0.49] | 0.13 | -0.04 | [-0.28, 0.20] | 0.75 |
| 5+ | -0.79 | [-3.06, 1.48] | 0.5 | -0.09 | [-0.35, 0.17] | 0.5 | 0.19 | [-0.19, 0.58] | 0.32 | -0.02 | [-0.29, 0.25] | 0.89 |
| Birthweight, kg |  |  |  |  |  |  |  |  |  |  |  |  |
| <2.5 | ref |  |  | ref |  |  | ref |  |  |  |  |  |
| ≥2.5 | 1.82 | [-0.50, 4.14] | 0.12 | 0.41 | [0.15, 0.67] | 0.002 | -0.1 | [-0.32, 0.11] | 0.34 | -0.1 | [-0.34, 0.14] | 0.42 |
| Mother’s age (at birth), y |  |  |  |  |  |  |  |  |  |  |  |  |
| <20 | ref |  |  | ref |  |  | ref |  |  |  |  |  |
| 20-29 | 1.69 | [-0.51, 3.89] | 0.13 | 0.05 | [-0.12, 0.22] | 0.56 | 0.15 | [-0.02, 0.32] | 0.082 | 0.1 | [-0.08, 0.28] | 0.29 |
| 30+ | 1.97 | [-0.38, 4.33] | 0.1 | 0.15 | [-0.15, 0.45] | 0.32 | -0.06 | [-0.38, 0.26] | 0.7 | 0.23 | [-0.06, 0.53] | 0.12 |
| Mother’s HIV status |  |  |  |  |  |  |  |  |  |  |  |  |
| Negative | ref |  |  | ref |  |  | ref |  |  |  |  |  |
| Positive pregnancy | -0.16 | [-1.86, 1.54] | 0.85 | -0.1 | [-0.26, 0.06] | 0.22 | -0.02 | [-0.25, 0.22] | 0.89 | -0.39 | [-0.59, -0.19] | <0.001 |
| Positive since pregnancy | -0.31 | [-2.15, 1.53] | 0.74 | 0.03 | [-0.17, 0.23] | 0.77 | 0.03 | [-0.11, 0.18] | 0.64 | -0.2 | [-0.35, -0.06] | 0.007 |
| **Current life factors** |  |  |  |  |  |  |  |  |  |  |  |  |
| Child hospitalizations (since birth) |  |  |  |  |  |  |  |  |  |  |  |  |
| 0 | ref |  |  | ref |  |  | ref |  |  |  |  |  |
| 1+ | -0.15 | [-1.64, 1.33] | 0.84 | 0.01 | [-0.16, 0.18] | 0.91 | 0.13 | [-0.16, 0.42] | 0.38 | -0.05 | [-0.29, 0.19] | 0.66 |
| Mother’s education |  |  |  |  |  |  |  |  |  |  |  |  |
| None/primary | ref |  |  | ref |  |  | ref |  |  |  |  |  |
| Some secondary or higher | 2.34 | [0.93, 3.75] | 0.0011 | 0.32 | [0.15, 0.49] | <0.001 | -0.11 | [-0.28, 0.06] | 0.21 | 0.05 | [-0.12, 0.22] | 0.57 |
| Maternal current BMI |  |  |  |  |  |  |  |  |  |  |  |  |
| <18.5 | -2 | [-7.62, 3.61] | 0.48 | -0.26 | [-0.69, 0.17] | 0.23 | -0.24 | [-0.42, -0.06] | 0.0077 | -0.06 | [-0.40, 0.28] | 0.72 |
| 18.5-24 | ref |  |  | ref |  |  | ref |  |  |  |  |  |
| 25-29 | 1.46 | [-0.09, 3.01] | 0.065 | 0.19 | [0.01, 0.37] | 0.041 | 0.08 | [-0.13, 0.28] | 0.47 | 0.01 | [-0.17, 0.18] | 0.95 |
| 30+ | 4.67 | [3.15, 6.19] | <0.001 | 0.71 | [0.53, 0.89] | <0.001 | -0.02 | [-0.20, 0.16] | 0.85 | 0.02 | [-0.16, 0.19] | 0.86 |
| Owns fridge |  |  |  |  |  |  |  |  |  |  |  |  |
| No | ref |  |  | ref |  |  | ref |  |  |  |  |  |
| Yes | 1.36 | [0.12, 2.59] | 0.031 | 0.3 | [0.15, 0.45] | <0.001 | 0.11 | [-0.03, 0.25] | 0.12 | 0.05 | [-0.09, 0.18] | 0.49 |
| Stunting |  |  |  |  |  |  |  |  |  |  |  |  |
| < 2.5 kg | ref |  |  |  |  |  |  |  |  |  |  |  |
| ≥ 2.5 kg | -0.02 | [-2.52, 2.47] | 0.98 |  |  |  |  |  |  |  |  |  |

OR indicates odds ratio; aOR indicates adjusted odds ratio; CI indicates confidence interval.

^1^ Overfat model also adjusted for child sex and age.
