# Supplementary material for: Breastfeeding, HIV exposure, childhood obesity, and prehypertension: A South African cohort study
Source: PLoS Med. 2019 Aug 27;16(8):e1002889. doi: 10.1371/journal.pmed.1002889 (PMC6711496; doi:10.1371/journal.pmed.1002889)
Supplement: S4 Table — BMI, body mass index. (DOCX) [file pmed.1002889.s005.docx]

**S4 Table.** Multivariable quantile regressions for sample percentiles at clinical thresholds of underfat (body fat percent) and underweight (BMI z-score) on maternal, child, and household early life and current factors, child ages 7-11 years.

|  | **Body fat % (n=1364)^1^** | | | **BMI z-score (n=1361)** | | |
| --- | --- | --- | --- | --- | --- | --- |
|  | **B** | **95% CI** | **p-value** | **B** | **95% CI** | **p-value** |
| Age stop any breastfeeding, mo |  |  |  |  |  |  |
| 0 | ref |  |  | ref |  |  |
| 1-5 | -0.26 | [-1.11, 0.59] | 0.55 | -0.6 | [-1.00, -0.21] | 0.0028 |
| 6-11 | -0.27 | [-0.71, 0.17] | 0.23 | -0.1 | [-0.44, 0.23] | 0.55 |
| 12+ | -0.59 | [-1.14, -0.04] | 0.036 | -0.57 | [-0.97, -0.18] | 0.0044 |
| **Early life factors** |  |  |  |  |  |  |
| Birth order |  |  |  |  |  |  |
| 1-2 | ref |  |  | ref |  |  |
| 3-4 | 0.22 | [-0.28, 0.72] | 0.39 | -0.09 | [-0.50, 0.32] | 0.66 |
| 5+ | -0.62 | [-1.30, 0.06] | 0.072 | -0.54 | [-1.31, 0.24] | 0.17 |
| Birthweight, kg |  |  |  |  |  |  |
| <2.5 | ref |  |  | ref |  |  |
| ≥2.5 | 1.65 | [1.07, 2.23] | <0.001 | 0.84 | [-0.84, 2.52] | 0.33 |
| Mother’s age (at birth), y |  |  |  |  |  |  |
| <20 | ref |  |  | ref |  |  |
| 20-29 | -0.85 | [-1.40, -0.30] | 0.0025 | -0.23 | [-0.63, 0.18] | 0.27 |
| 30+ | -1.03 | [-1.80, -0.26] | 0.0084 | -0.33 | [-0.81, 0.16] | 0.19 |
| Mother’s HIV status |  |  |  |  |  |  |
| Negative | ref |  |  | ref |  |  |
| Positive pregnancy | 0.19 | [-0.32, 0.70] | 0.47 | -0.03 | [-0.36, 0.30] | 0.85 |
| Positive since pregnancy | -0.14 | [-0.72, 0.44] | 0.64 | -0.33 | [-0.77, 0.11] | 0.14 |
| **Current life factors** |  |  |  |  |  |  |
| Child hospitalizations (since birth) |  |  |  |  |  |  |
| 0 | ref |  |  | ref |  |  |
| 1+ | 0.25 | [-0.33, 0.83] | 0.4 | -0.26 | [-0.67, 0.15] | 0.21 |
| Mother’s education |  |  |  |  |  |  |
| None/primary | ref |  |  | ref |  |  |
| Some secondary or higher | 0.33 | [-0.06, 0.72] | 0.098 | 0.21 | [-0.12, 0.55] | 0.2 |
| Maternal current BMI |  |  |  |  |  |  |
| <18.5 | -0.97 | [-1.52, -0.42] | <0.001 | -0.01 | [-1.63, 1.60] | 0.99 |
| 18.5-24 | ref |  |  | ref |  |  |
| 25-29 | 0.18 | [-0.27, 0.63] | 0.43 | 0.13 | [-0.41, 0.68] | 0.64 |
| 30+ | 1.79 | [1.23, 2.35] | <0.001 | 0.74 | [0.18, 1.30] | 0.0097 |
| Owns fridge |  |  |  |  |  |  |
| No | ref |  |  | ref |  |  |
| Yes | 1.01 | [0.65, 1.37] | <0.001 | 0.01 | [-0.33, 0.36] | 0.95 |
| Stunting |  |  |  |  |  |  |
| < 2.5 kg | ref |  |  |  |  |  |
| ≥ 2.5 kg | -0.21 | [-0.94, 0.52] | 0.57 |  |  |  |

OR indicates odds ratio; aOR indicates adjusted odds ratio; CI indicates confidence interval.

^1^ Underfat model also adjusted for child sex and age.
